# Analytical methods for structural ensembles and dynamics of intrinsically disordered proteins

**DOI:** 10.1007/s12551-016-0234-6

**Published:** 2016-11-22

**Authors:** Marieke Schor, Antonia S. J. S. Mey, Cait E. MacPhee

**Affiliations:** 1 School of Physics and Astronomy, University of Edinburgh, Edinburgh, UK; 2 School of Chemistry, University of Edinburgh, Edinburgh, UK

**Keywords:** Intrinsically disordered proteins, Conformational ensembles, Enhanced sampling simulations, Markov state modeling

## Abstract

Intrinsically disordered proteins, proteins that do not have a well-defined three-dimensional structure, make up a significant proportion of our proteome and are particularly prevalent in signaling and regulation. Although their importance has been realized for two decades, there is a lack of high-resolution experimental data. Molecular dynamics simulations have been crucial in reaching our current understanding of the dynamical structural ensemble sampled by intrinsically disordered proteins. In this review, we discuss enhanced sampling simulation methods that are particularly suitable to characterize the structural ensemble, along with examples of applications and limitations. The dynamics within the ensemble can be rigorously analyzed using Markov state models. We discuss recent developments that make Markov state modeling a viable approach for studying intrinsically disordered proteins. Finally, we briefly discuss challenges and future directions when applying molecular dynamics simulations to study intrinsically disordered proteins.

## Introduction

For roughly a century, the central dogma of structural biology has been that proteins need to fold into a well-defined three-dimensional structure in order to function (Fisher 1894). However, over the past two decades, it has become clear that many proteins contain long disordered regions or are completely disordered under physiological conditions (Wright and Dyson 2015; Dyson and Wright 2005; Tompa 2005; Dunker et al. 2002; Uversky 2002; Tompa 2011). Such intrinsically disordered proteins (IDPs) and intrinsically disordered regions (IDRs) are found across all four kingdoms but are particularly abundant in eukaryotes, where they play crucial roles in processes like signaling and regulation (Dyson and Wright 2005). For example, the majority of eukaryotic transcription factors fall in this category and as such it is not surprising that they play a major role in human disease, including neurodegenerative diseases and many cancers (Uversky et al. 2008; Iakoucheva et al. 2002). For the remainder of this review, we will use the term IDP to refer to either a fully intrinsically disordered protein or a disordered region studied on its own.

**Fig. 1:**
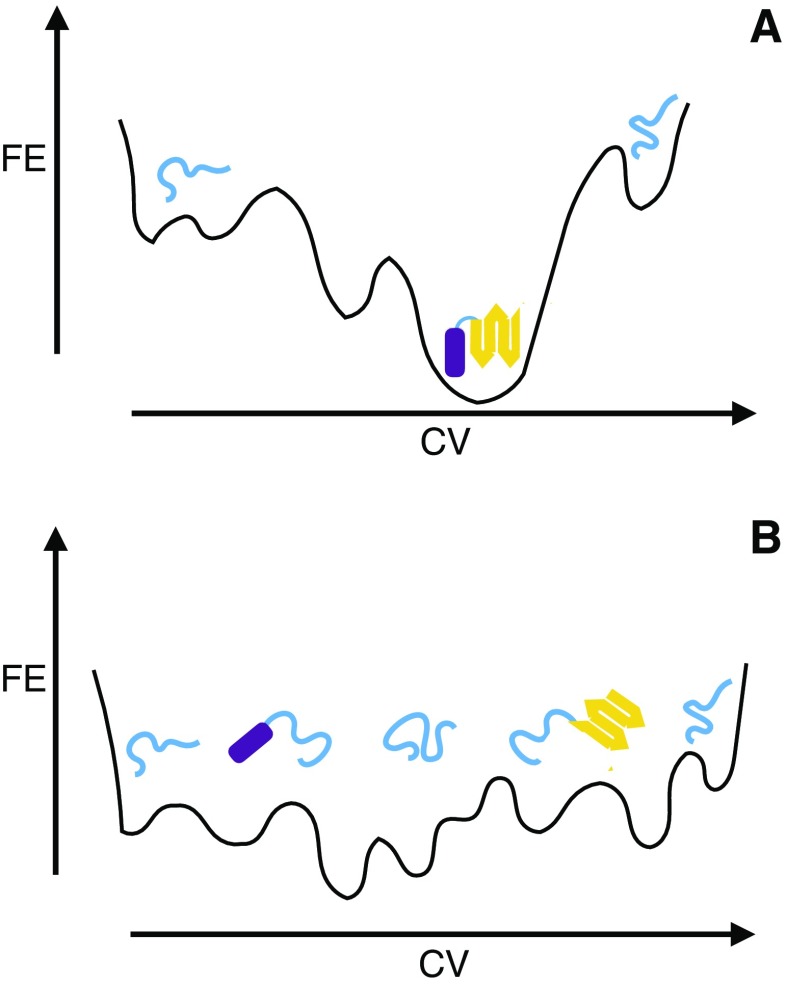
Free-energy landscapes of folded and intrinsically disordered proteins. **a** The free-energy landscape of folded proteins typically has a funnel-like shape with the overall minimum corresponding to the folded structure. **b** In contrast, intrinsically disordered proteins have rough free-energy landscapes with many local minima and no dominant overall minimum

Rather than encoding for a well-defined, energetically stable three-dimensional structure, the free-energy landscapes of IDPs are comparatively flat, allowing the protein to sample many different conformations (Fig. 1) (Jensen et al. 2014). Perhaps counterintuitively, many IDPs are promiscuous binders that are able to interact with high specificity, but low affinity, with multiple biological targets (Kovacs et al. 2013; Wright and Dyson 2015; Boehr et al. 2009). In order to understand how the primary sequence encodes the diverse mechanisms characteristic for this class of proteins, it is essential to obtain atomic resolution descriptions, which capture both structures and dynamics of IDPs in their free and complexed states.

Since classical methods in structural biology, particularly crystallography, have been developed to study the dominant, stably-folded conformation, they are inherently unsuitable to study IDPs. However, solution-state NMR (see Jensen et al. 2014) for an excellent review of recent developments in NMR spectroscopy) and molecular dynamics simulations hold great potential to characterize the conformational ensemble of IDPs. Computer power, quality of protein force fields, and simulation code have all increased significantly in recent years. As such, atomistic molecular dynamics (MD) simulations have become an established technique for studying protein folding and the underlying free-energy landscapes.

In this review, we will discuss recent developments in advanced MD simulation methodologies and their application to IDPs. We will first discuss popular methods, originally developed to study folding of ordered proteins, which have been successfully applied to characterize the structural ensemble of IDPs, along with some recent applications. As dynamics within the conformational ensemble is another key characteristic of IDPs, a substantial part of this review deals with rigorous analysis strategies to obtain this information from molecular dynamics simulations. Finally, we will briefly discuss current limitations in using simulations to characterize IDPs and outline promising developments for future studies.

## Characterizing the structural ensemble of IDPs

Even with current computer power and improved molecular simulation codes, obtaining adequate sampling remains an issue in atomistic simulations in explicit solvent. This is particularly true for IDPs, with their high conformational heterogeneity. Several methods have been developed to overcome this sampling issue and these methods can be divided into two groups: unbiased (e.g., temperature replica exchange, solute tempering) and biased (e.g., umbrella sampling, metadynamics) enhanced sampling simulations. The ensemble of conformations generated with these methods is typically analyzed by clustering the data based on structural similarities or calculating the probability of observables. Here we will discuss the strengths and weaknesses of several replica exchange and metadynamics-based implementations, which have been used extensively to characterize ordered proteins, when applied to IDPs. The application of another enhanced sampling method, multicanonical ensemble MD, to study IDPs has recently been reviewed elsewhere (Ikebe et al. 2016).

### Replica exchange

**Fig. 2:**
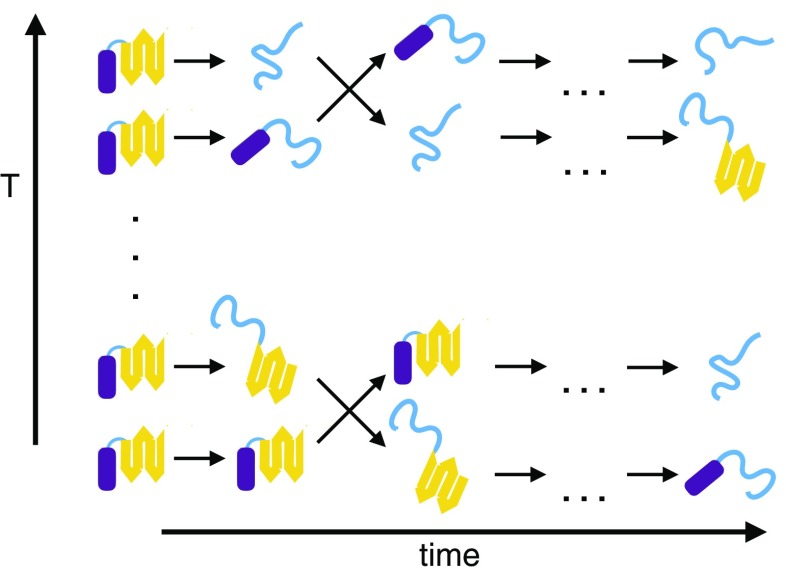
Schematic of replica exchange simulations. Multiple copies (replicas) of the system are simulated in parallel at different temperatures. Crossing free-energy barriers is facilitated at higher temperature and hence many different conformations are explored. At set time intervals, exchanges between neighboring replicas are attempted. If the exchange is accepted, the higher temperature conformation cannotequilibrate at a lower temperature. This cycle of simulating and exchanging is repeated many times, such that replicas move up and down in temperature several times, ultimately leading to an equilibrated conformational ensemble at the temperature of interest

In a temperature replica exchange MD (tREMD) simulation (Sugita and Okamoto 1999), multiple copies (replicas) of the system are simulated in parallel, all at different temperatures (see Fig. 2). At least one of the replicas should be at the temperature of interest and at least one should be at a temperature high enough to rapidly overcome free-energy barriers between metastable states. The high-temperature replica allows for fast sampling of conformational space and, as exchanges between replicas adjacent in temperature take place, the conformational ensemble at the temperature of interest can be built. The exchanges are governed by a Monte Carlo scheme and detailed balance is ensured by applying the Metropolis rule:

$$P(acc) = \min(1, \exp(\delta \beta \delta E)), $$

where *d* *ß* is the difference in inverse temperature and *d* *E* is the difference in potential energy between the replicas.

The tREMD method is routinely available in popular biomolecular simulation packages, including NAMD (Phillips et al. 2005), AMBER (Salomon-Ferrer et al. 2013), and GROMACS (Abraham et al. 2015). tREMD simulations are particularly useful as an explorative tool to efficiently sample the free-energy landscape and as such have seen much use in characterizing the conformational ensemble of IDPs. So far, tREMD simulations have primarily been used to characterize the conformational ensemble of IDPs in the unbound state.

One of the first applications of tREMD to study IDPs highlighted the importance of transient formation of secondary structure in the A *ß*40 and A *ß*42 peptides (Sgourakis et al. 2007). These peptides both form amyloid fibrils found in plaques in the brains of patients with Alzheimer’s disease. A *ß*42 is far more aggregation-prone and neurotoxic than A *ß*40. The extensive sampling provided by the tREMD simulations shows that the conformational ensemble of A *ß*42 is much more diverse and, contrary to A *ß*40, transient secondary structure formationis largely found in the C-terminus of the peptide, emphasizing the importance of the two extra C-terminal residues. Similarly, transient formation of secondary structure elements has also been demonstrated for the highly flexible N- or C-terminal protrusions of histone proteins, where states with high secondary structure content may be important for interaction with linker DNA (Potoyan and Papoian 2011). In this latter study, 50–54 replicas, depending on system size, spanning a temperature range of 300-450 K, were simulated for 55–60 ns/replica.

As many IDPs are structured when bound to their interaction partners, the observation of transient secondary structure formation raises the question of whether the binding partner selects and stabilizes a conformation transiently sampled by the IDP in isolation. Support for this mechanism of binding came from extensive tREMD simulations of the NCBD protein in implicit (Zhang et al. 2012) and explicit (Knott and Best 2012) solvent. This nuclear coactivator binding domain of the transcriptional coactivater CBP is disordered in solution but folds into a triple helix when bound to its binding partner ACTR. The more accurate explicit solvent simulations used 48 replicas to span a temperature range of 304–424 K with a simulation time of 250 ns/replica. In agreement with experimental data, a large proportion of residual helical structure was found in the unbound ensemble, particularly in the two terminal helices. The middle helix is observed less frequently and not in conjunction with the terminal two helices, pointing to a mechanism that is part conformational selection and part induced folding upon interaction with ACTR.

However, the residual structure observed in tREMD simulations does not always coincide with the structure observed in the bound conformation. Miller et al. studied the conformational ensemble of several IAPP variants using 40 replicas to span a temperature range of 300–575 K with a simulation time of 200 ns/replica (Miller et al. 2013). The human IAPP peptide (hIAPP) forms *ß*-sheet-rich amyloid fibrils but in solution tREMD simulations, a relatively high *a*-helical content was noted. Notably, the degree of residual helical structure observed for variants of the peptide correlated with the aggregation propensity, with less aggregation-prone variants having more structural flexibility (Miller et al. 2013).

The flexible nature of IDPs makes them highly amenable to regulation via post-translational modifications, including phosphorylation and glycosylation (Babu et al. 2011; Xie et al. 2007). tREMD simulations have been used to investigate how such modifications affect the conformational ensemble of IDPs. Zerze and Mittal studied the effect of O-linked glycosylation on the conformational ensemble of the tau _174-183_ fragment and the hIAPP peptide (Zerze et al. 2015). For the tau peptide, 24 replicas in the temperature range of 300–545 K were simulated for 100 ns/replica. For the larger hIAPP peptide, a total of 40 replicas were used to cover the 300–500 K temperature range (150 ns/replica). This study found only a mild effect of glycosylation on the conformational ensemble of the tau _174-183_ fragment and the hIAPP peptide (Zerze et al. 2015). In contrast, phosphorylation of Ser133 of the KID peptide leads to a significant redistribution of helical substates and is likely to affect recognition of its binding partner KIX (Ganguly et al. 2009). This implicit solvent simulation study employed 12 replicas in the range of 270–500 K with a simulation time of 200 ns/replica.

Although tREMD has proven very useful in analyzing protein conformational space, and there are many variants of tREMD as discussed in a recent review (Ostermeir and Zacharias 2013), a major limitation is its poor scaling with system size as the number of replicas needed increases as $\mathcal {O}(f^{1/2})$, where *f* is the system’s total number of degrees of freedom. The reason for this poor scaling can be understood directly from the probability of accepting exchanges $\exp (\delta \beta \delta E)$ between adjacent replicas. As systems get larger, this rule dictates smaller spacing in temperature in order to ensure viable acceptance probabilities and thus more replicas are needed to cover the same temperature range. Several methods have been developed to overcome this limitation. Here, we will briefly discuss Hamiltonian replica exchange (hREMD) (Fukunishi et al. 2002) and solute tempering (Liu et al. 2005; Wang et al. 2011).

### Hamiltonian replica exchange

Although tREMD is the most commonly used implementation, other replica coordinates can be used to modify the underlying energy surface. In principle, any coordinate can be used as long as detailed balance is obeyed by employing the metropolis acceptance criterion. The replica coordinate can be coupled to the Hamiltonian, or force field, of the system in a scheme that is generally referred to as Hamiltonian replica exchange MD (hREMD) (Fukunishi et al. 2002). Suitable replica coordinates facilitate backbone structural transitions by scaling the strength of, for instance, hydrogen bonds or hydrophobic interactions. The scaling is done such that the interactions are weaker for consecutive replicas, such that refolding transitions are fast at the most downscaled replica. Typically, fewer replicas are needed in hREMD than in tREMD to obtain similar conformational sampling.

### Solute tempering

Another method developed to overcome poor scaling with system size in conventional tREMD simulation is the replica exchange with solute tempering (REST) method (Liu et al. 2005). In a REST simulation, the system is divided into two parts, with one acting as a bath and remaining at the temperature of interest and the other part (usually the whole protein, although part of the protein, or the protein and solvation shell waters, could be used instead) is effectively heated up. As only energy differences arising from protein and protein–water interaction but not water–water interactions, contribute to the acceptance probability, the number of replicas needed to cover a certain temperature range is significantly reduced. However, REST does not perform well for large systems involving sizable conformational changes (Huang et al. 2007).

Several groups have independently combined the core concept of REST, dividing the system into a cold and a hot part, with the idea of scaling Hamiltonians of hREMD (Moors et al. 2011; Terakawa et al. 2011; Wang et al. 2011). For the hot part of the system, the electrostatic, Lennard–Jones and proper dihedral terms (the force-field parameters contributing to energy barriers) are scaled such that the interactions inside this part are kept at an effective temperature of *T*/*?*. Interactions within the cold part are kept at temperature *T* and interactions between the cold and hot parts are kept at an intermediate temperature $T/\sqrt {\lambda }$. This method, often referred to as REST2, has been implemented in GROMACS (Terakawa et al. 2011; Bussi 2014).

The improved efficiency of REST2 makes this an attractive tool for studying the conformational ensemble sampled by IDPs. A recent application of this method to the disordered N-terminal fragment of the nacre protein allowed a reduction of almost a factor of six in the number of replicas needed to span the required temperature range compared to conventional tREMD (Brown et al. 2014). The n16 nacre protein is a framework protein associated with biogenic mineral stabilization in the Japanese pearl oyster. Its 30 residue N-terminus (n16N) is essential for the stabilization of the mineral component in nacre and is largely disordered. REST2 simulations of n16N in its apo- and Ca ^2+^-complexed forms, using 16 replicas to span a temperature range of 300–500 K, support the hypothesis that the peptide can be divided into three subdomains. The N-terminal subdomain and the central amyloid-like domain (SD1 and SD2) feature stabilization through intrapeptide aromatic contacts. The C-terminal subdomain (SD3) has a higher charge density and shows more structural flexibility. This domain is likely to play a crucial role in capturing Ca ^2+^, whereas SD1 and SD2 are essential for the formation of interpeptide contacts and hence multipeptide complexes (Brown et al. 2014). REST2 was also used to characterize the conformational space of *Helicobacter pylori* UreG (Musiani et al. 2013), a class of intrinsically disordered enzymes involved in the maturation of the urease virulence factor in bacterial pathogens. The same protocol was applied to HypB, a protein from *Methanocaldococcus jannaschii* that is closely related in sequence and function but has not been classed as an IDP. A total of 24 replicas were needed to span a temperature range of 300 to 450 K, compared to an estimated 100 replicas if conventional tREMD had been used. The authors found that the regions involved in catalysis show substantial structural rigidity. In contrast to HypB, the regions in UreG that are involved in interaction with metallochaperones to form multiprotein complexes are more unfolded (Musiani et al. 2013).

It should be noted that although the hREMD method is powerful in enhancing backbone transitions (Ostermeir and Zacharias 2013), to the best of our knowledge, non-REST2 flavors of hREMD have not yet been successfully applied to characterize IDPs. We do, however, believe that these will prove useful in future work in this field, particularly in analyzing binding and unbinding effects of IDPs and binding partners as protein–protein interactions can be used as replica coordinates.

### Metadynamics

**Fig. 3:**
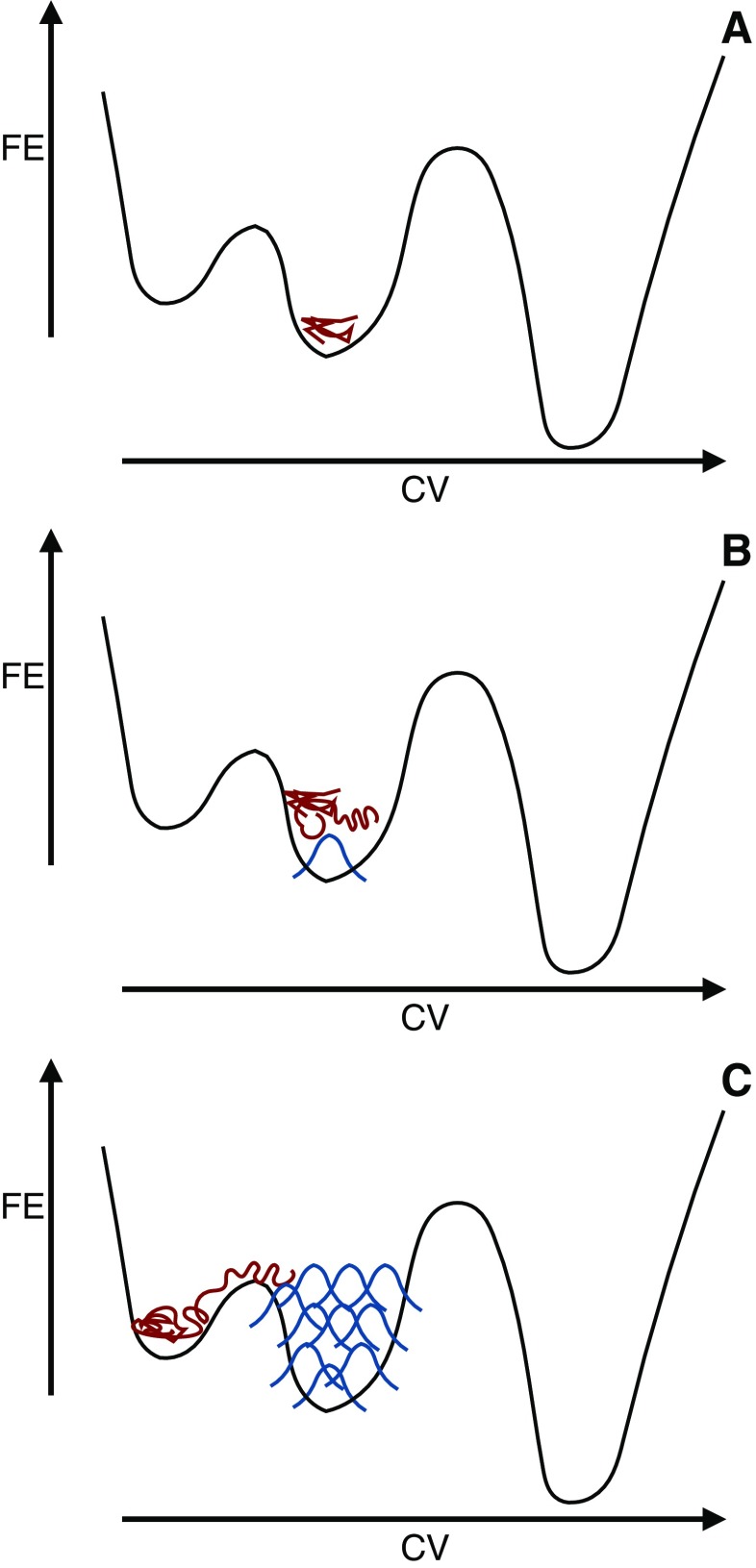
Metadynamics simulations. **a** A simulation is started in one of the free-energy minima along the chosen collective variable. **b** After a set time, *t*, during which the system explores locally, a Gaussian is deposited to discourage revisiting this area.**c** After some time N*t, the local minimum has been filled with Gaussians, and a new region of phase space is explored by the simulation. Ultimately, this region will also be filled and the higher barrier will be crossed

Enhanced sampling methods that employ a biasing potential are often considerably cheaper in terms of computer time than replica exchange-based approaches. Out of the biased enhanced sampling methods, metadynamics-based approaches appear the most suitable for exploring the conformational ensembles sampled by IDPs. Similar to, for instance, conformational flooding (Grubmüller 1995) and local elevation methods (Huber et al. 1994), in a metadynamics simulation the system is discouraged from visiting previously explored regions by a biasing potential (Laio and Parrinello 2002). This history-dependent biasing potential is built by periodically depositing Gaussians along the trajectory of the collective variable (CV) (see Fig. 3)

1 $$ V_{G}(s(r), t) = w \sum\limits_{t^{\prime} =\tau_{G}, 2\tau_{G}, ... t^{\prime}<t} \exp \frac{(s(r)-s(t^{\prime}))^{2}}{2{\sigma_{s}^{2}}},  $$

where *s*(*r*) is the CV as a function of the atomic coordinates, *w* and *s* are the height and width of the Gaussians and *t*_*G*_ is the rate at which they are deposited. Ultimately, when the simulation reaches equilibrium, the biasing potential should exactly compensate the unbiased free-energy profile along the chosen CV. Assuming a perfect choice of CV, the accuracy of the method depends wholly on the height and width of the Gaussians and the frequency at which these are deposited.

A major advantage of metadynamics over methods such as umbrella sampling (Torrie and Valleau 1977) and steered MD (Park and Schulten 2003) is that no a priori knowledge of the end states is required and that multiple CVs can be used. However, the efficiency of metadynamics scales poorly with the number of CVs used and in practice is limited to three CVs. Moreover, in practical applications, the free-energy profile does not converge to a definite value but rather fluctuates around the correct result. There is also a significant risk of pushing the system into physically irrelevant regions of phase space. Hence, several adaptations to the scheme have been proposed. Here, we will discuss the popular well-tempered (Barducci et al. 2008; Bonomi et al. 2009) and bias-exchange methods (Piana et al. 2007). All of these versionsof metadynamics are available in the PLUMED plug-in (Tribello et al. 2014), which interfaces with many popular simulation packages.

#### Well-tempered metadynamics

Well-tempered metadynamics (WTM) was developed to address issues of poor convergence and risk of sampling outside the physically relevant phase space in standard metadynamics simulations. In this method, the height of the Gaussians is not fixed but scaled, ensuring dampening of the biasing potential towards the exact result in the limit of long simulations.

WTM has been applied to characterize binding of the IDPs PTMA and NRF2 to the Kelch domain of Keap1 (Do et al. 2015). Interaction with NRF2 is crucial for the regulation of cellular responses to oxidative stress. Although there is a high degree of sequence similarity between the Kelch binding domains of PTMA and NRF2, the affinity of PTMA is approximately 100 times weaker. Two 3 *µ*s well-tempered metadynamics simulations using three CVs were run with either PTMA or NRF2 and the Kelch domain. Multiple binding and unbinding events were observed for both systems. NRF2’s higher affinity for the Kelch domain may be explained by the observation that PTMA is much more disordered than NRF2 (Do et al. 2015). In its unbound state, NRF2 has a tendency to form short hairpin structures, supporting the hypothesis of coupled folding and binding for the NRF2-Kelch complex.

#### Bias-exchange metadynamics

Bias-exchange metadynamics combines the strengths of metadynamics and replica exchange (Piana et al. 2007). In this method, several metadynamics simulations of the system are run in parallel, all being biased in independent CVs. It is crucial here to include one ’neutral’ replica, which does not experience a biasing potential. Exchanges between the systems are attempted periodically in a replica exchange-type fashion.

This method has successfully been applied to investigate the mechanisms by which the oncoprotein c-Myc is inhibited by a small drug molecule (Michel and Cuchillo 2012). Seven biased replicas (with different CVs) and one neutral replica were simulated for 120 ns (per replica) to generate the unbound (Apo) ensemble. It should be noted that the CVs used here were general, rather than optimized specifically for this system. One more replica was added for the bound (Holo) state biased in a CV accounting for ligand interaction. The authors show that the ligand-binding domain of c-Myc can bind the ligand in multiple distinct conformations. Interestingly, many of these conformations are also wholly or partially present in the unbound ensemble, providing support for a conformational selection mechanism (Michel and Cuchillo 2012).

#### Comparing sampling efficiencies of metadynamics-based approaches

The sampling efficiencies of unbiased MD, bias-exchange metadynamics and well-tempered metadynamics simulations have recently been compared for a 20-residue disordered peptide derived from the Neh2 domain of the Nuclear factor erythroid 2-related factor 2 (Nrf2) protein (Do et al. 2014). The authors compared conformational ensembles obtained from 3 *µ*s unbiased MD simulation, 3 *µ*s well-tempered metadynamics simulation using two CVs and a 460-ns bias exchange metadynamics simulation with eight replicas (seven biased and one neutral) and validated their results against X-ray crystallography and NMR spectroscopy data. Although both metadynamics protocols significantly enhance sampling, the bias-exchange scheme proved far more effective than well-tempered metadynamics (Do et al. 2014). General CVs, like *ß*-sheet content and number of hydrogen bonds, were used and uniformly applied to all Neh2 residues. As such, no prior knowledge of experimentally observed structures is necessary and they do not bias towards a limited set of predefined structures.

Similarly, the sampling efficiency of temperature replica exchange MD and bias-exchange metadynamics simulations have been assessed for the IDP hIAPP (Zerze et al. 2015). Forty replicas, spanning the 300–575 K temperature interval, were used for the tREMD simulation, with a simulation time of 200 ns/replica (8 *µ*s cumulative simulation time). The bias-exchange metadynamics simulation employed seven biased replicas and a neutral replica with a simulation time per replica of 650 ns (5.2 *µ*s cumulative simulation time). The free-energy profiles and secondary structures populated in the two ensembles obtained with the two approaches are very similar. However, bias-exchange metadynamics explores larger regions of conformational space with less (cumulative) simulation time, suggesting that this method is computationally more efficient. The authors do note that bias-exchange metadynamics simulations are less straightforward to set up as the choice of CVs needs to be validated (Zerze et al. 2015).

## Understanding dynamics within the structural ensemble

So far, we have focused on methods to characterize the structural ensemble of IDPs. It would, however, be desirable to not just know which structures can be adopted by a particular IDP but also to analyze how these structures interconvert. This sort of information will be particularly valuable when assessing the effect of post-translational modifications and small molecule binding. Over the past 15 years, Markov state models (MSM) have become increasingly popular for rigorously analyzing biomolecular simulation data with the aim of understanding long time-scale dynamics. MSMs have been used extensively to study protein folding. One of the earliest examples looked at *ß*-hairpin formation in the Trp zipper (Singhal et al. 2004). Since then, MSMs have been used to gain insight into the folding pathways of larger systems including PinWW (Noé et al. 2009), MR121-GSGS-W peptide (Noé et al. 2011), folding of FiP35 WW domain, GTT, NTL9, and protein G (Beauchamp et al. 2012). More recently, more complicated processes like allostery (Malmstrom et al. 2015), protein ligand binding (Plattner and Noé 2016; Doerr and Fabritiis 2014), and amyloid fibril formation (Schor et al. 2015) have all been studied with the help of MSMs.

The recently developed transition-based reweighting analysis method (TRAM) is likely to greatly enhance the use of MSMs to analyze the structural dynamics of IDPs. As discussed in “?? Characterizing the structural ensemble of ?? IDPs”, one often has to rely on enhanced sampling methods to achieve the required sampling. While these methods are very useful in speeding up estimations of equilibrium properties, information on the unbiased, room-temperature dynamics are lost. However, it has recently been shown that it is in fact possible to combine data from various different enhanced sampling methods (Wu et al. 2016), such as replica exchange or umbrella sampling with non-biased trajectories to improve accuracy in the estimation of both thermodynamic and dynamic properties. A family of these estimators has been discussed in the literature (Mey et al. 2014; Wu et al. 2014; Wu and Noé 2014; Wu et al. 2016) and TRAM is the most general version. It is statistically optimal and does not rely on binning of energies, cf. binning energies in the weighted histogram analysis method (WHAM) for stationary properties. Instead, it estimates a multi-ensemble Markov model that allows the extraction of full thermodynamic and kinetic information from all thermodynamic ensembles (Wu et al. 2016). The choice of thermodynamic ensemble would be open to the simulator but could for example be the different temperatures of a tREMD simulation. The advantage of using TRAM is that it does not rely on rate models, and only uses the fact that it is possible to reweight configurations between ensembles while adhering to detailed balance. TRAM has been implemented in the MSM analysis package pyEMMA (Scherer et al. 2015).

### Background of Markov state modeling

MSMs assume that the dynamics of e.g., a protein or protein ligand complex can be seen mathematically as a stochastic process. There are two assumptions made, (a) it is possible to find a low-dimensional discrete projection onto a set of coordinates, often referred to as clustering and (b) the dynamics coming from a Markov jump process between these discrete states capture features of the high-dimensional protein dynamics well. The mathematical object that describes the jump process is a transition matrix $\mathbf {T}(\tau )\in \mathbb {R}^{N\times N}$ that contains the conditional probabilities of going between *N* discrete states, or microstates, *i* and *j*. In other words, *T*_*ij*_ contains the probability of being in a state *i* at time *t* and jumping to state *j* at *t* + *t*.

2 $$ T_{ij}(\tau) = P(x_{t+\tau}=j, x_{t}=i). $$

The lagtime *t* dictates at what time interval the transition matrix is constructed from the discretized trajectory data and needs to be adjusted to ensure a memoryless jump process between the microstates. By definition, the transition matrix is a stochastic matrix that can be used to extract both stationary and dynamic properties of the system, described by the eigenvalues and eigenvectors. The stationary probability of each of the microstates is contained in the eigenvector of the transition matrix that corresponds to eigenvalue *?*_1_=1, given by:

3 $$ \boldsymbol{\pi}^{T}\mathbf{T} = \boldsymbol{\pi}^{T}. $$

In contrast, timescales and associated processes are associated with all other eigenvalues and eigenvectors of the transition matrix.

4 $$ \mathbf{T}{\psi}_{i} =\psi_{i}\lambda_{i}, $$

where *?*_*i*_ is the i ^th^ right eigenvector of the transition matrix. Equally, a set of left eigenvectors *?*_*i*_ can be defined. It is usually possible to observe a gap in the eigenvalue spectrum, revealing dominant slow processes in the system of interest. The associated timescale to these slow processes is related to the eigenvalues and a relaxation timescale or implied time scale, *t*_*i*_ can be defined as:

5 $$ t_{i} = \frac{-\tau}{\ln|\lambda_{i}|}.  $$

**Fig. 4:**
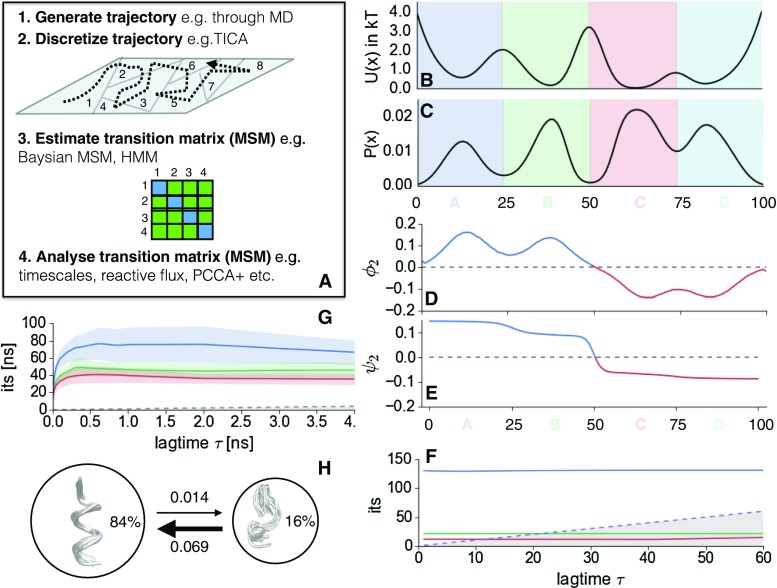
**a** Summary of steps needed for MSM construction and analysis. **b** Toy potential consisting of 100 discrete states. **c** Stationary distribution corresponding to the toy potential. **d** Second left eigenvector estimated from a Brownian dynamics simulation sampling from **b**. **e** Second right eigenvector estimated from MSM. **f** Three dominant relaxation timscales estimated from the MSM; errors computed using a Bayesian MSM are smaller than the width of the lines. **g** Implied timescales with bootstrapping for a real-world toy system: deca-alanine simulated with the CHARMM27 force field. **h** Coarse-grained two-state model for deca-alanine using the hidden Markov model approach. Structures shown are the extrema of the left eigenvectors of the microstate MSM. MSM estimation was done using the software pyEMMA (Scherer et al. 2015)

The inverse of the implied timescale can be seen as a transition rate for the given process. Various older (Prinz et al. 2011; Pande et al. 2010) and more recent reviews (Chodera and Noé 2014) and a book (Bowman et al. 2013) discuss the theoretical aspects of MSMs in great detail. To facilitate the construction of MSMs, different software is available to go from the raw MD trajectory data to a comprehensive MSM description, such as EMMA/pyEMMA (Senne et al. 2012; Scherer et al. 2015) and MSMBuilder (Beauchamp et al. 2011). Typically, a few steps have to be taken to be able to construct an MSM with either of the available software packages. A pictorial summary of these steps can be found in Fig. 4a, which involves clustering the data, followed by estimating a transition matrix from the clustered data and then analyzing this transition matrix, which forms the heart of the MSM. There are different ways to analyze the transition matrix, but usually a mixture of further coarse graining the states to get a transition network on a coarse set of states or using transition path theory for computing most likely fluxes in the transition network defined by the transition matrix are common approaches. A typical analysis is shown in summary in Fig. 4b–f for a four-well toy potential (b). Using a Brownian dynamics simulation, a single particle is used to sample the toy potential. The corresponding stationary distribution is shown in (c), directly computed from the potential. The trajectory is discretized into 100 bins, as indicated by the *x*-axis tick labels and a transition matrix is computed from the discrete trajectories. Figure 4d and e show the second left and right eigenvectors of the transition matrix, respectively, and it becomes obvious that the slowest process of the system is crossing the barrier at bin 50, as seen by the sign change in the eigenvectors. Furthermore, it can be observed that the left eigenvector is the right eigenvector simply weighted by the stationary distribution. The associated timescale *t*_*i*_, often called the implied or relaxation timescale, stemming from the Brownian dynamics and computed according to Eq. 5, is shown in Fig. 4f for different lag times. In an ideal situation, the implied timescale does not depend on the lagtime unless the process has already decayed. In real systems, such a flat implied timescale is only achieved when increasing the lagtime (Fig. 4h). For the final estimation of the transition matrix, usually a lagtime for which the dominant slow processes are lagtime-independent is chosen and a Bayesian MSM estimation is the preferred option, as this also allows the estimation of error bars on observables of interest (Trendelkamp-Schroer et al. 2015). For the four-well potential, a coarse graining into four macrostates would be the preferred coarse representation of the system, but was omitted here. Figure 4 g and h show the implied timescales for the MSM of deca-alanine and the resulting coarse-grained two-state model using the hidden Markov Model approach as implemented in pyEMMA (Scherer et al. 2015; Noé et al. 2013). Details of the simulation using the CHARMM27 forcefield can be found in reference (Vitalini et al. 2015).

### Application of MSMs to IDPs

As MSMs give insight into both the structural ensemble and the conformational kinetics, they are particularly useful when comparing the effect of, for instance, post-translational modifications. Comparison of MSMs built for the IDP kinase-inducible domain (KID) and its phosphorylated form (pKID) has identified a metastable, partially ordered state with at least a 60-fold decrease in the rate of conformational exchange in the phosphorylated case (Stanley et al. 2014). As such, phosphorylation kinetically locks a region of the peptide, in this case the region that interacts with the binding partner. Kinetic locking of binding regions would have a major effect on the affinity of an IDP for its partner.

Given their success in the field of protein folding, MSMs have so far seen surprisingly few applications in the field of IDPs. A major reason for this is that the first step in constructing an MSM is to define a good set of microstates through clustering of the data, and this is challenging for IDPs. It is critical that the clustering reflects structures that are kinetically separated. Traditionally, backbone root-mean- square deviation (RMSD) has been used for clustering. Although this tends to give reasonable results for many folded-protein systems, RMSD-based clustering is not suitable for IDPs, as in these highly flexible systems states that have a high RMSD may rapidly interconvert, and conversely, states that have a low RMSD may be kinetically separated. Recently, two approaches have been developed to solve this issue, time-lagged independent component analysis (TICA) (Pérez-Hernndez et al. 2013; McGibbon and Pande 2013; Noé and Clementi 2015) and the variational approach (Nüske et al. 2014; Noé and Nüske 2013).

#### Time-lagged independent component analysis (TICA)

The TICA approach is similar in nature to principle component analysis (PCA) in that it uses linear combinations of sets of coordinates to achieve a dimensional reduction of the original high-dimensional MD trajectory (Pérez-Hernndez et al. 2013; McGibbon and Pande 2013; Noé and Clementi 2015). Here, not only spatial variance in the coordinates is taken into account but also the kinetic distance, trying to maximize kinetic distance in order to capture the slowest process of the system the best. This means that typically a dimensional reduction using TICA is done first on a set of relevant coordinates, such as distances. This is then followed by clustering on these reduced coordinates, at which point the usual route for MSM construction and analysis can be continued.

This method has been shown to be very effective at capturing relevant kinetics in the 30 residue intrinsically disordered peptide derived from the kinase-inducible domain (KID) in its phosphorylated state (Pérez-Hernndez et al. 2013). Clustering the data based on the ten dominant TICA coordinates enabled the authors to reveal five dominant transitions in the system, three of which could already be resolved using just four TICA coordinates. TICA-based clustering also enabled MSM building for the C-terminal, cytoplasmic tail of the human cluster determinant 4 (CD4) protein, which interacts with two viral accessory proteins of the HIV-1 virus (Ahalawat et al. 2015). TICA was used to project the high-dimensional simulation data onto five dominant dimensions. Analysis of the resulting MSM indicated that the peptide has an almost flat free-energy landscape with transiently populated secondary structures and rapidly interconverting metastable states.

#### Variational approach to conformation dynamics

A downside of the TICA-based clustering approach is that it is fairly ad hoc and depends heavily on the order parameters fed into the TICA algorithm. Another recent approach borrows ideas from quantum chemistry in order to avoid a discrete clustering, as done in the MSM, all together. This is a variational approach that tries to approximate the eigenfunctions of the dynamic process describing the protein dynamics directly by using the method of linear variation, in which a Roothan–Hall-type generalized eigenvalue problem can be formulated to optimally describe the eigenvalues and eigenvectors of the propagator^1^ of the dynamics (Nüske et al. 2014; Noé and Nüske 2013). The advantage is that the user can now choose an appropriate basis set, making the discretization a data-driven process, which can lead to fewer basis functions needed than when using a crisp MSM discretization. In fact, an MSM discretization can be seen as a special case of the variational approach, where the basis functions are step functions. Furthermore, if basis functions are chosen with a certain chemical intuition, the interpretation of the estimation of the eigenfunction is much easier than in the case of the MSM. The drawback is that if the protein system is large, the possible combination of basis functions becomes prohibitively large. Recently, a way of identifying a good way of combining basis functions has been proposed (Nüske et al. 2016). This uses a sparse tensor product approach that allows description of high-dimensional eigenfunctions with a small set of eigenfunctions, making it possible to also tackle larger molecular systems.

While this method has yet to be applied to IDPs, it is likely that this systematic way to discretize the simulation data will greatly help in future applications.

## Challenges and future

Ongoing developments in enhanced sampling simulation methodologies and analysis methods, along with better hardware, mean that MD simulations have been recognized as a powerful tool for characterizing structural ensembles of IDPs. However, the fact that we can now reach the required time- and length scales has highlighted some issues with commonly used biomolecular force fields (Best et al. 2014; Piana et al. 2015; Rauscher et al. 2015; Vitalini et al. 2015). Particular issues are that certain force fields overstabilize secondary structure elements, potentially leading to overrepresentation of certain conformations in the unbound ensemble, and many produce structural ensembles that are on average too compact (Best et al. 2014; Fluitt and de Pablo 2015; Hoffmann et al. 2015; Pantelopulos et al. 2015; Piana et al. 2015; Rauscher et al. 2015). This is perhaps not surprising, as current force fields have primarily been developed to capture the structure and dynamics of folded proteins. As more and more experimental data comes available for IDPs, significant strides are being made to improve and validate biomolecular force fields in order to capture both folded and disordered proteins (Best et al. 2014; Piana et al. 2015).

In this review, we have focused on simulation strategies that generate structural ensembles de novo. Many of the studies highlighted here compared simulated ensembles to NMR data. Several methods have been developed recently that can use experimental data to guide the simulations. Two metadynamics-based approaches use NMR chemical shifts to this end. In replica-averaged metadynamics (RAM) simulations, the underlying force field is modified in a system-dependent manner, based on comparison of back-calculated experimental observables and actual experimental data (Camilloni et al. 2013; Camilloni and Vendruscolo 2014). As such, the experimental data are used to constrain the replica average. Another approach uses the chemical shifts as collective variables in a bias-exchange metadynamics setup (Gratana et al. 2013). Although NMR chemical shifts were used in both of these examples, it should be noted that it is in principle possible to use other experimental observables provided they can be accurately back-calculated from the simulations. As NMR is a very powerful experimental tool to study IDPs, these methods are expected to find extensive use in characterizing structural ensembles as well as aid force-field optimization for this class of proteins.

## Footnotes

1 The propagator is simply the transpose of the non-discrete version of the transition matrix, i.e., the transfer operator, and has the same eigenfunctions as the transfer operator.
